# Perceived social support and diet quality among ethnic minority groups in Yunnan Province, Southwestern China: a cross-sectional study

**DOI:** 10.1186/s12889-021-11787-5

**Published:** 2021-09-23

**Authors:** Qiang Zhang, Yuan Ruan, Wenmin Hu, Juanjuan Li, Jiang Zhao, Min Peng, Rong Wan, Xiangdong Min, Shaomei He, Zhitao Liu

**Affiliations:** 1 Department of Nutrition and Food Hygiene, Yunnan Center for Disease Control and Prevention, Kunming, 650022 China; 2 Department of Chronic Disease Prevention, Lanping County Center for Disease Control and Prevention, Lanping, 671400 China

**Keywords:** Ethnic minority, Diet balance index, Social support, China

## Abstract

**Background:**

Social support is an important health determinant and may affect dietary behaviors. The purpose of this study was to examine the relations between perceived social support and the Chinese Diet Balance Index-16 (DBI-16) among ethnic minority groups in Southwest China.

**Methods:**

This cross-sectional study was conducted between May 2019 and August 2020 among six ethnic minority groups native to Yunnan Province (*n* = 3564). Perceived social support from family, friends and significant others were measured with the Multi-dimensional Scale of Perceived Social Support (MSPSS). Dietary data were obtained using a 100-item Food Frequency Questionnaire (FFQ) and a lifestyle questionnaire. Lower Bound Score (LBS), Higher Bound Score (HBS) and Diet Quality Distance (DQD) which represent inadequate, excessive and unbalanced food intake respectively were calculated to measure the compliance with the recommendations of the Dietary Guidelines for Chinese 2016.

**Results:**

One thousand four hundred ninety-six men and two thousand sixty-eight women were included. 51.2% of the subjects had moderate or high levels of inadequate intake; 21.3% had moderate or high levels of excessive intake; and 74.0% had moderate or high levels of unbalanced dietary intake. With potential confounders adjusted, support from family was negatively associated with inadequate intake, while support from friends was positively associated with inadequate and excessive intake. No significant associations were found between perceived social support from significant others and diet quality indicators.

**Conclusions:**

An unbalanced diet is common among adults of the ethnic minority groups in Yunnan Province, Southwest China. Social support should be taken into account in designing nutrition interventions rather than focusing solely on individuals.

**Supplementary Information:**

The online version contains supplementary material available at 10.1186/s12889-021-11787-5.

## Background

Unhealthy eating has become a serious public health problem worldwide [1, 2]. It is estimated that 11 million deaths worldwide (22% of all deaths among adults) were attributable to dietary risk factors such as high intake of sodium, and low intake of whole grains and fruits [3]. Over the past decades, China has also experienced an accelerating nutrition transition characterized by unhealthy changes in dietary patterns and increasing prevalence of diet-related diseases [4, 5]. Nearly half of Chinese adults are facing the problem of double burden of malnutrition, which refers to the coexistence of micronutrient deficiencies along with overweight and obesity within individuals [6].

To promote healthy eating behaviors among the public, some countries have developed dietary guidelines based on scientific evidence and local dietary habits [7, 8]. Dietary indexes such as Healthy Eating Index (HEI) for Americans and Diet Balance Index (DBI) for Chinese, have also been constructed to measure how well individual diets meet recommendations of the guidelines [9, 10]. In addition to dietary assessment, these priori indexes are also used to examine associations between diet quality and health outcomes [11, 12]. Notably, data from the 2010–2012 China Nutrition and Heath Surveillance showed that 73.6% adults were at moderate or high levels of inadequate food intake, while 27.9% were at moderate or high levels of excessive food intake [13]. What’s more, diet imbalance is more likely to be found among people living in rural areas, with lower education level and household income [12]. In recent years, social and interpersonal influences on dietary behaviors have received increasing attention. For example, a study in Canada shows that social deprivation (an indicator reflects deprivation of social networks, from family to community) is associated with lower diet quality [14]. In contrast, another study from Switzerland reported that women who dined more often with healthy eaters were on a higher diet quality and a lower body mass index (BMI) [15]. These findings suggest that social networks may have a shared perception of healthy eating, which would influence individual’s dietary practices. Thus, individual’s social relationships would be an important consideration for healthy eating promotion.

Social support is the emotional, instrumental and informational aid exchanged through social relationships and interpersonal transactions, which can be measured as perceived support or received support [16]. Perceived social support is the mostly measured index for its ease of measurement and strong associations with health outcomes [17, 18]. Although positive and causal relations between social support and health have been well documented, the influence of social support on some key health-related behaviors like diet are still understudied, especially in adults. For single food intakes of individuals, social support often appears a protective effect. For example, two studies from the United States show that social support was correlated with high consumptions of fruits and vegetables, or low consumptions of sugar and processed meat [19, 20]. In contrast, the relations between social support and overall diet quality remain controversial. In one study, social support was positively associated with HEI-2010 among middle-aged and older US men; however, such association was not statistically significant among American minority youths [21, 22]. Moreover, sources of social support may have different effects on eating behaviors. For instance, fruit and vegetable consumption were positively associated with family support, but not with friend or pastor support among African-Americans [23]. In addition, effects of social support on dietary behaviors may also differ in gender, age and ethnicity [24, 25]. Thus, although social support being perceived as one of the best strategies to promote health, the associations of between social support and dietary behaviors need to be further examined in certain contexts.

Yunnan Province is a less developed and multi-ethnic province in Southwest China. Benefited from the National Poverty Alleviation and Development Program, the local economy has been growing at a high speed in the past decades, especially in the ethnic regions. Therefore, we hypothesize that unbalanced dietary consumptions would have emerged among the ethnic minority groups and social support may have a role in this process. In his study, we examined the associations between sources of perceived social support and DBI-16 among adults of six ethnic minority groups in Yunnan Province. The purpose of the study was to evaluate dietary intakes of the ethnic groups and further understand the influential factors.

## Methods

### Study design

Data for this study were from the 2019–2020 nutrition and health survey of ethnic minorities native to Yunnan Province. Adults of six ethnic minority groups with a total population less than 150,000 were included, which were A Chang, Bu Lang, De Ang, Jing Po, Ji Nuo and Pu Mi [26]. Most of these groups live in certain mountain towns. Thus, a multistage sampling method was used to recruit participants. First, six towns with the largest population of A Chang, Bu Lang, De Ang, Jing Po, Ji Nuo or Pu Mi ethnicity were chosen as the study sites. Second, two villages were selected with the Probability Proportional to Size sampling method (number of households). Then, 150 households were randomly selected based on local household registration information. Finally, all adults at home of the sampled households were invited to join the survey, except those who were on a prescribed diet or who had serious illnesses.

**Fig. 1:**
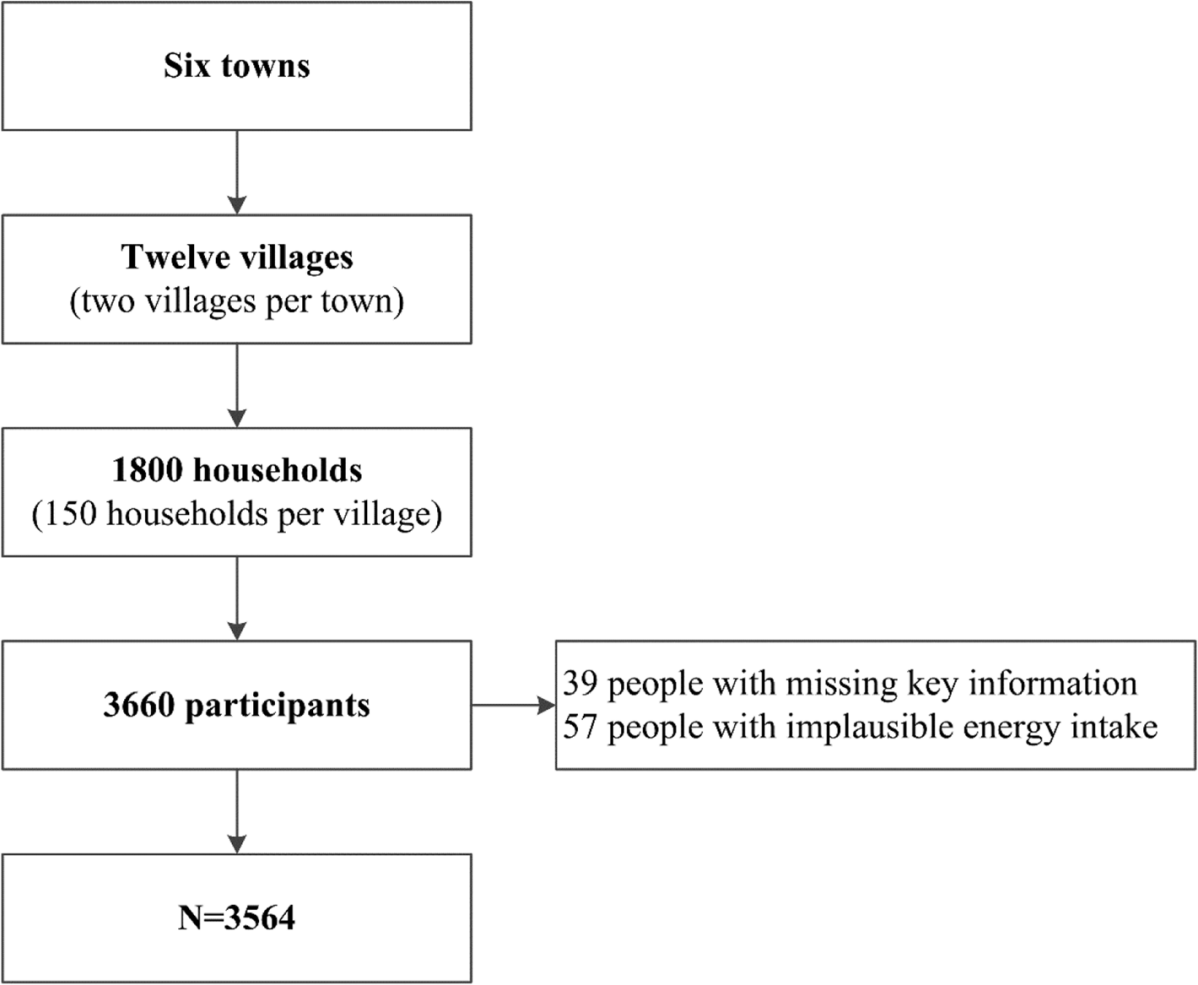
Flowchart of the study population

The sample size was determined from careful power analysis considering the following four factors: a prevalence of hypertension of 15% (the most prevalent nutrition-related disease in the population), a desired precision of 3%, a type I error of 0.05 and the population of each ethnic minority group. The minimum sample size per minority group ranged from 545 to 570. We aimed at recruiting 600 participants per site, considering potential of 10% non-response. Data were collected through a face-to-face interview by trained local health workers. Altogether, 3660 participants aged 18 years and over completed the survey, with a response rate of 89.0%. Ninety-six participants were excluded because of missing key information (39 subjects), or implausible energy intake (< 800 kcal (kcal) per day or > 6000 kcal for men and < 600 kcal or > 4000 kcal for women, 57 subjects) [27]. The final ana1ysis has been conducted on 3564 participants (Fig. 1).

### Dietary data collection

Dietary intakes over the past year were assessed using a 100-itemFood Frequency Questionnaire (FFQ). These items include cereals (9 items), beans (7 items), fresh vegetables (8 items), salt vegetables (2 items), mushrooms (2 items), algae (2 items), fruits (7 items), dairy (7 items), meat (14 items), fish (12 items), eggs (3 items), snacks (18 items) and beverages (9 items), which were commonly consumed by the Chinese population. The FFQ was validated and proved to be a useful tool for the collection individual dietary intake in face-to-face interviews [28, 29]. Based on the frequency and amount of food consumption reported by individual participants, daily intake of each food item was calculated.

In addition, data of alcohol consumption was derived from the lifestyle questionnaire of the survey. Participants were inquired the drinking frequency and quantity of alcoholic beverages (such as high alcohol liquor, low alcohol liquor, wine, beer and etc.) in the past 12 months. Alcohol in each alcoholic beverage was converted following DBI-16 criteria and then summed to calculate average alcohol consumption per day Daily energy intake for individuals was calculated using the dietary data in conjunction with the China Food Composition Table [30]. Moreover, daily intakes of the food items were also summed by food group classifications correspond with the DBI-16 criteria for further analysis [31].

### Dietary balance Index-16

DBI-16 is devised to assess diet quality in Chinese population. It comprises eight components from “the Chinese Dietary Guidelines 2016” including: (1) Cereals; (2) Vegetables and fruits; (3) Milk and dairy products, soybean and soybean products; (4) Animal foods; (5) empty energy foods; (6) Condiments; (7) Diet variety; (8) Drinking water. A score of 0 for each DBI-16 component means meeting the recommended intake amounts. Positive scores (range 0–12) were used to assess excessive intake levels of empty energy foods and condiments, which the guidelines recommended consuming in a “limited” amount. Similarly, negative scores (range − 12, 0) were used to assess inadequate intake levels of vegetables and fruits, dairy and soybeans, dietary diversity and drinking water, which the guidelines recommended that people should “eat plenty” or “enjoy”. Moreover, both positive and negative scores were used to evaluate the intake levels of cereals (− 12, 12) and animal food (− 12, 8), which the guidelines recommend consuming in an “appropriate” amount. In considering the difference of nutrient requirements in energy consumption, the scoring of each component is based on eleven energy intake levels, respectively. The intakes of 12 identified food groups are used to assess the diet variety component of DBI-16. These food groups were (1) rice and products; (2) wheat and products; (3) corn, coarse grains and products, starchy roots and products; (4) dark-colored vegetables; (5) light-colored vegetables; (6) fruits; (7) soybean and products; (8) milk and dairy products; (9) red meat and products; (10) poultry and game; (11) eggs; (12) fish and shellfish. A score of 0 is assigned to a food group if the intakes reach or exceed the lowest recommended intake (5 g for soybean and 25 g for other 11 food groups). Else, a score of − 1 is assigned. The score of diet variety component ranges from − 12 to 0 [24, 29, 30]. Scoring details of DBI-16 can be found in Table S1.

Based on the scores for each DBI-16 component, three indicators of diet quality are calculated. Higher Bound Score (HBS), the indicator for excessive intake, is calculated by adding all the positive scores. Lower Bound Score (LBS), the indicator for inadequate intake, is computed by adding all the negative scores. Diet Quality Distance (DQD), indicator of unbalanced food intake, is calculated by the absolute values of both positive and negative scores. For data on water consumption was not available in the survey, the component of ‘drinking water’ was not assessed in this study. Therefore, the range of HBS, LBS and DQD were: 0 to 44, 0 to 60 and 0 to 84, respectively. Each indicator is further divided into five levels to reflect diet quality for simplicity. They are “no problem” (a score of 0), “almost no problem” (less than 20% of the total score), “low level” (between 20 and 40% of the total score), “moderate level” (between 40 and 60% of the total score) and “high level” (greater than 60% of the total score) [24, 29, 30].

### Measurements of perceived social support

Perceived social support was measured by using the Multi-dimensional Scale of Perceived Social Support (MSPSS). This scale consists of 12 items with 4 items measuring family support, 4 items measuring friend support and 4 items measuring significant others support. Responses for each item were ranked on a five-point Likert scale. Total scores for family, friend and significant others support were respectively calculated and used in following analysis. MSPSS is one of the most extensively used instruments to assess social support and the reliability and validity have verified in different groups. Correlation analysis showed that the alpha coefficient of the scale was 0.95 in this study. In this study, confirmatory factor analysis showed a robust three-factor structure of the scale and the Cronbach’s alpha coefficients of three factors were 0.94, 0.91 and 0.95, respectively. In addition, the alpha coefficients for A Chang, Bu Lang, De Ang, Jing Po, Ji Nuo and Pi Mi ethnicity ranged from 0.93 to 0.95. The MSPSS can be found in Table S2.

### Covariates

Sociodemographic characteristics of the participants were incorporated in the analyses as potential confounders, including age, sex, ethnicity, education, income, and Engel’s coefficient. Age was divided into five groups (18–34, 35–44, 45–59 and ≥ 60 years) in the descriptive statistics, while treated as a continuous variable in the logistic regression analyses. Education was classified as three levels (primary school and below, middle school, high school and above). Income was measured with household income per capita in the last 12 months and categorized into four categories (< 5000 Yuan, 5000–9999 Yuan and ≥ 10,000 Yuan). Engel’s coefficient was classified as four levels (≥0.50 subsistence level; 0.40–0.49 well level; 0.30–0.39 relatively rich; < 0.30 rich) [32].

### Data analysis

Categorical variables (e.g., sex, and age groups) are presented as percentage, while numeric variables which normally distributed (e.g., perceived social support scores) were presented as mean and standard deviation (SD) and which not normally distributed were presented as median (e.g., DBI-16 component scores and indicators). Kruskal-Wallis one-way analysis was used to examine the associations between sociodemographic characteristics and DBI-16 indicators. Logistics regression analysis was used to estimate the associations between perceived social support scores and diet quality with potential confounders adjustment. All statistical analyses were performed with SAS Software 9.4 (SAS Institute, Cary, North Carolina, USA). Statistical significance was set at *P* < 0.05.

## Results

Table 1 shows the characteristics of the study population. A total of 3564 ethnic adults were included in the final analysis, comprising 1496 men (42%) and 2068 women (58%). Proportions of subjects aged 18–34, 35–44, 45–59, 60 years and older were 20.2, 21.7, 35.6 and 22.5%, respectively. Each of the six ethnic minority groups approximately accounted for 16% of the sample. Most of the participants were farmers and over one third only had primary school or no formal education. Nearly one fourth of the adults had a household income less than 5000 Yuan per year.

**Table 1:** Characteristics of the participants

	N	%
Sex (%)		
Men	1496	42.0
Women	2068	58.0
Age in years (%)		
18–34	721	20.2
35–44	774	21.7
45–59	1268	35.6
≥ 60	801	22.5
Ethnicity (%)		
A Chang	592	16.6
Bu Lang	604	16.9
De Ang	611	17.1
Jing Po	606	17.0
Ji Nuo	598	16.8
Pu Mi	553	15.5
Education (%)		
Primary school and below	1248	35.0
Middle school	1423	39.9
High school and above	893	25.1
Occupations (%)		
Farmer	3364	94.4
Others	200	5.6
Household income per capita (%)		
< 5000 Yuan/year	870	24.4
5000–9999 Yuan/year	1367	38.4
≥ 10,000 Yuan/year	1327	37.2
Engel’s coefficient (%)		
≥ 0.50	867	24.3
0.40–0.49	684	19.2
0.30–0.39	1087	30.5
< 0.30	926	26.0

Table 2 presents the scores for DBI-16 components and the percentage of people with each score. Over intakes of cereals, meat and oil were common, with the score for about 80 to 90% participants being in the positive range. In contrast, under intakes of dairy, fruits and fish were also common, with over 90% of the ethnic adults having a negative score. Diet variety was very poor in the population, with almost all of them (99.9%) in the negative range.

**Table 2:** DBI-16 component scores and percentage of the participants with each score

Scores	Cereals	Vegetables & Fruits		Dairy & Beans		Animal source foods			Oil & alcohol		Sugar& salt		Diet variety
		Vegetables	Fruits	Dairy	Beans	Meat	Fish	Eggs	Oil	Alcohol	Sugar	Salt	
−12 ~ −11													0.2
−10 ~ −9													3.6
−8 ~ −7	0.1												26.5
−6 ~ −5	0.9	13.2	35.3	84.4	19.0								42.4
−4 ~ −3	1.4	47.6	44.0	10.7	32.3	1.4	75.4	33.1					20.6
−2 ~ −1	2.9	25.8	11.7	3.2	17.0	7.8	15.3	35.4					6.7
0	6.2	13.4	8.9	1.7	31.7	9.9	9.3	14.7	15.8	86.7	99.8	23.8	0.1
1 ~ 2	7.7					14.7		7.6	32.6	7.4	0.1	56.5	
3 ~ 4	11.9					66.2		9.2	23.1	2.1	0.1	15.7	
5 ~ 6	14.4								28.5	3.8	0	4.0	
7 ~ 8	14.6												
9 ~ 10	9.7												
11 ~ 12	30.2												
Median	7	−3	−4	−5	−3	4	−4	−2	3	0	0	1	−6

The distribution of DBI-16 indicators is presented in Table 3. The LBS indicated that 51.2% of the participants had moderate or high levels of inadequate food intake, respectively. The distribution of HBS indicated that 21.3% of people had a moderate or excessive food intake. According to the distribution of the DQD, an indicator used to evaluate the overall unbalance in dietary intake levels, 74% of the ethnic adults had moderate or high levels of unbalanced food intake.

**Table 3:** Distribution of DBI-16 indicators among the participants

Indicators	Range	Distribution of Diet Quality (%)			
		Almost no problem	Low level	Moderate level	High level
LBS	0–44	0–8 (3.8)	9–17 (45.0)	18–26 (50.0)	26–44 (1.2)
HBS	0–60	0–11 (9.5)	12–23 (69.2)	24–35 (21.1)	36–60 (0.2)
DQD	0–84	0–16 (0.8)	17–33 (25.2)	34–50 (66.5)	51–84 (7.5)

Table 4 shows the median scores for LBS, HBS and DQD by sociodemographic characteristics of the participants. Results of Kruskal-Wallis test indicated that women and people who were younger, had higher education or income were less likely to have inadequate, excessive and unbalanced dietary intakes.

**Table 4:** Predictors of DBI-16 indicators

	LBS	*p*	HBS	*p*	DQD	*p*
Sex						
Men	20		16		40	
Women	19	< 0.01	15	< 0.01	40	0.04
Age in years						
18–34	18		15		38	
35–44	20		15		40	
45–59	20		15		41	
≥ 60	21	< 0.01	16	< 0.01	42	< 0.01
Education						
Primary school and below	20		16		41	
Middle school	20		16		41	
High school and above	19	< 0.01	15	< 0.01	38	< 0.01
Annual Income (Yuan)						
< 5000	20		16		42	
5000–9999	20		16		41	
≥ 10,000	19	< 0.01	15	< 0.01	39	< 0.01

Associations between moderate or high levels of inadequate and excessive food intake and perceived social support are shown in Table 5. Logistic regression models with potential confounders adjustment showed that support from family were negatively associated with inadequate food intake, while support from friends were positively associated with both inadequate and excessive food intake. No significant associations were found between support from significant others and DBI-16 indicators.

**Table 5:** Associations between moderate or high levels of inadequate/excessive food intake and social support

Dependent variables		Perceived social support from		
		Family	Friends	Significant others
LBS	Model 1	−0.02 (− 0.05, 0.01)	**0.04 (0.01, 0.07)**	0.02 (− 0.01, 0.05)
	Model 2	**−0.04 (− 0.06, − 0.01)**	**0.04 (0.01, 0.08)**	0.01 (− 0.02, 0.04)
HBS	Model 1	− 0.01 (− 0.05, 0.02)	**0.06 (0.02, 0.10)**	0.01 (− 0.03, 0.05)
	Model 2	− 0.02(− 0.05, 0.02)	**0.05 (0.01, 0.10)**	0.02 (− 0.02, 0.06)

Model 1: Crude model; Model 2: adjustment for age, sex, education, income, ethnicity and Engel’s coefficient. Values in bold are statistically significant at the 0.05 level

Table 6 show the correlation coefficients between DBI-16 component scores and perceived social support. Support from family were positively associated with intakes of fruits, dairy, fish, egg and diet variety, while negatively associated with vegetable and meat consumption. Support from friends were positively associated with intakes of dairy, beans, fish, egg, sugar and diet variety, while negatively associated with cereals, vegetable and meat consumptions. Support from significant others were positively associated with intakes of dairy, beans, fish, egg, sugar and diet variety, while negatively associated with cereals, vegetable and meat consumptions.

**Table 6:** Correlations between DBI-16 component scores and perceived social support

DBI component	Score range	Social support from		
		Family	Friends	Others
Cereals	−12, 12	−0.01	**−0.10**	**− 0.07**
Vegetables	−6, 0	**−0.13**	**− 0.19**	**− 0.19**
Fruits	− 6, 0	**0.10**	− 0.01	0.02
Dairy	−6, 0	**0.10**	**0.13**	**0.14**
Beans	−6, 0	0.03	**0.15**	**0.14**
Meat	− 4, 4	**−0.12**	**− 0.18**	**− 0.04**
Fish	− 4, 0	**0.09**	**0.15**	**0.16**
Eggs	−4, 4	**0.05**	**0.13**	**0.15**
Oil	0, 6	−0.01	**0.05**	0.03
Alcohol	0, 6	0.02	0.01	0.01
Sugar	0, 6	0.02	**0.05**	**00.7**
Salt	0, 6	0.01	−0.01	0.01
Diet variety	−12, 0	**0.07**	**0.16**	**0.17**

Values in bold are statistically significant at the 0.05 level

## Discussions

The cross-sectional study examined the associations between diet quality and perceived social support among adults of six ethnic minority groups in Yunnan Province, Southwest China.. Dietary assessment with DBI-16 indicated that dietary imbalance is common among the population, especially among men and those who are older, had lower education or income levels. Notably, support from family, friends and significant others showed different effects on healthy eating. To our best knowledge, this was the first study to explore interpersonal factors of dietary behaviors among ethnic minorities in less developed areas of China.

There are 15 ethnic minority groups native to Yunnan Province. Historically, these groups live in remote, poor areas. A study conducted in Yunnan among preschool-aged children 10 years ago showed that the prevalence of stunting, underweight and wasting were significantly higher in ethnic minority groups than in Han ethnicity [33]. Benefited from the poverty alleviation work in recent years, these areas have achieved impressive economic developments. In the former case, changes in lifestyle and diet often come with rapid economic developments [34, 35]. A longitudinal study in China showed that between 1995 and 2014 stunting, thinness continued to reduce in school-aged children and adolescents while the prevalence of obesity markedly increased [36]. In this study, Engel’s coefficient is 0.4, suggesting that living standard of the ethnic minority groups is just at a crucial stage of nutrition transition. Besides, most of the ethnic adults are low educated and have poor health awareness, which would make them difficult to develop or keep a healthy dietary habit in the nutrition transition. A recent study showed that although the prevalence of non-communicable chronic diseases in adults of Nu ethnicity was still lower than in Han Chinese, excessive intakes of meat and cereals were more prevalent in the former [37, 38]. For developing and implementing targeted intervention programs, it is of significance to evaluate diet quality and influential factors of the population.

Our findings indicate that 51.2% of the ethnic minority groups had moderate or high levels of inadequate food intake; 23.3% had moderate or high levels of excessive food intake; and 74.0% had moderate or high levels of unbalanced food intake. In contrast to a recent Chinese national representative study, diet quality of the study population already exceeded than the rural residents but was still inferior to the urban residents (LBS: 56.9–91.0%; HBS: 22.3–33.7%; DQD: 58.6–89.1%) [31]. Diet quality is usually better among urban residents due to higher income and more diversified food supply [39]. This study reflects the effect of rapid urbanization on dietary patterns among the population. Our results that there were socioeconomic disparities in diet quality are consistent with previous studies, highlight the necessity of paying more attention on people with low socioeconomic status [10, 40]. One interesting finding of our study is that only 10% of the study population consumed adequate vegetables and fruits, which is lower than people in Shanghai (about 20%) [39]. But actually, vegetables and fruits are fairly cheap and widely available in the ethnic areas. In contrast, over 80% of the subjects had excessive intake of meat, which was even higher than urban residents in Shanghai (about 60%) [39]. These findings suggest that the ethnic minority groups would not have formed a correct perception of what constitutes a healthy diet. Thus, more research is required to understand the influential factors in addition to economic development and personal income.

In the literature, social support has been proposed as a social psychological mechanism that social ties affect bodily and emotional well-being [41]. People can obtain both normative and behavioral guidance through comparisons with others, for example, norms about healthy eating. Besides, facts or recommendations from others may enable subsequent behavioral changes that make everyday tasks more efficient, economical or successful. Thus, social support is recognized as a promising strategy to promote health and has been used in clinical and adolescent nutrition interventions [42, 43]. In this study, social support from different sources showed different effects on healthy eating. Family support had a protective effect for inadequate food intake, whereas the support from friends was a risk factor for both inadequate and excessive food intake. And no significant associations were found between diet quality and the support from significant others. These results suggest that the influences of social relationships on dietary behaviors in the ethnic population are mainly from family and friends. In terms of individual food intakes, effect directions of the three sources of social support are generally consistent. This finding may imply that the perception of healthy eating across the three types of people are homogenous. In addition, the effects of perceived social support on individual food consumption are not completely consistent with recommendations of the Dietary Guidelines. For example, social support is positively associated with diet variety and dairy consumption but also negatively associated with vegetable intakes. This result may reflect the fact that nutrition knowledge among the ethnic minority groups are fragmental and inaccurate. In the current “Healthy China Action”, supportive environment construction (including infrastructure and relationships) is particularly emphasized [44, 45]. Thus, future studies may wish to focus on how to incorporate social support in healthy eating promotions of the public.

There are several limitations to this study. First, the cross-sectional design of the study restricts casual inference between dietary intakes and perceived social support. Second, although a FFQ could prevent seasonal variations in dietary intakes, the self-report food intake within a year might be subject to recall bias. Third, although socioeconomic factors are adjusted in the regression models, potential factors at contextual levels for both diets and social support are not fully considered, for example cultural differences in the ethnic minority groups. Last but not least, we only calculated the individual intake of condiments and sugar based on consumption at home and exclude the intakes outside the home. Thus, the consumption of oil, salt and sugar might be underestimated.

## Conclusions

In conclusion, unbalanced dietary consumption is common among the ethnic minority groups in Yunnan Province. Perception of support from family, friends and significant others has an effect on overall diet quality, and should be taken into account when designing and implementing intervention programs.

## Supplementary Information

**Additional file 1: Table S1.** Components of DBI-16.

**Additional file 2: Table S2.** The Multi-dimensional Scale of Perceived Social Support (MSPSS).

## Data Availability

The data used in this study are available from the corresponding author on reasonable request.

## Abbreviations

DBI-16: Diet Balance Index-16
LBS: Lower Bound Score
HBS: Higher Bound Score
DQD: Diet Quality Distance
MSPSS: Multi-dimensional Scale of Perceived Social Support
