# Supplementary material for: Perceived social support and diet quality among ethnic minority groups in Yunnan Province, Southwestern China: a cross-sectional study
Source: BMC Public Health. 2021 Sep 23;21:1726. doi: 10.1186/s12889-021-11787-5 (PMC8459473; doi:10.1186/s12889-021-11787-5)
Supplement: Supplementary file 2 — Additional file 2: Table S2. The Multi-dimensional Scale of Perceived Social Support (MSPSS). [file 12889_2021_11787_MOESM2_ESM.docx]

Table S2 The Multi-dimensional Scale of Perceived Social Support (MSPSS)

1. My family really tries to help me. 1 2 3 4 5

2. I get the emotional help & support I need from my family. 1 2 3 4 5

3. I can talk about my problems with my family. 1 2 3 4 5

4. My family is willing to help me make decisions. 1 2 3 4 5

5. My friends really try to help me. 1 2 3 4 5

6. I can count on my friends when things go wrong. 1 2 3 4 5

7. I have friends with whom I can share my joys and sorrows. 1 2 3 4 5

8. I can talk about my problems with my family. 1 2 3 4 5

9. There is a special person who is around when I am in need. 1 2 3 4 5

10. There is a special person with whom I can share joys and sorrows. 1 2 3 4 5

11. I have a special person who is a real source of comfort to me. 1 2 3 4 5

12. There is a special person in my life who cares about my feelings. 1 2 3 4 5

Note: 1. Disagree; 2. Mild disagree; 3. Neutral; 4. Mildly agree; 5. Agree.
